# The Crucial Role of SlGSNOR in Regulating Postharvest Tomato Fruit Ripening

**DOI:** 10.3390/ijms25052729

**Published:** 2024-02-27

**Authors:** Zesheng Liu, Dengjing Huang, Yandong Yao, Xuejuan Pan, Yanqin Zhang, Yi Huang, Zhiqi Ding, Chunlei Wang, Weibiao Liao

**Affiliations:** College of Horticulture, Gansu Agricultural University, 1 Yinmen Village, Anning District, Lanzhou 730070, China; lzs0724@163.com (Z.L.); huangdj3032@163.com (D.H.); yyd614636237@163.com (Y.Y.); panxj@st.gsau.edu.cn (X.P.); 17393139598@163.com (Y.Z.); huangyi202309@163.com (Y.H.); 15693198831@163.com (Z.D.); wangchunlei@gsau.edu.cn (C.W.)

**Keywords:** *S*-nitrosoglutathione reductase, tomato, postharvest storage, fruit ripening, softening, nutrients, nitric oxide

## Abstract

*S*-nitrosoglutathione reductase (GSNOR) is a well-known regulator in controlling protein *S*-nitrosylation modification and nitric oxide (NO) homeostasis. Here, a GSNOR inhibitor N6022 and *SlGSNOR* silencing were applied to investigate the roles of SlGSNOR in tomato fruit postharvest ripening. We found that the application of N6022 and *S*-nitrosoglutathione (GSNO, a NO donor), and *SlGSNOR* silencing delayed the transition of fruit skin color by improving total chlorophyll level by 88.57%, 44.78%, and 91.03%, respectively. Meanwhile, total carotenoid and lycopene contents were reduced by these treatments. Concurrently, the activity of chlorophyll biosynthesis enzymes and the expression of related genes were upregulated, and the transcript abundances of total carotenoid bioproduction genes were downregulated, by N6022 and GSNO treatments and *SlGSNOR* silencing. In addition, fruit softening was postponed by N6022, GSNO, and *SlGSNOR* silencing, through delaying the decrease of firmness and declining cell wall composition; structure-related enzyme activity; and gene expression levels. Furthermore, N6022, GSNO, and *SlGSNOR* silencing enhanced the accumulation of titratable acid; ascorbic acid; total phenol; and total flavonoid, but repressed the content of soluble sugar and soluble protein accompanied with the expression pattern changes of nutrition-related genes. In addition, the endogenous NO contents were elevated by 197.55%; 404.59%; and 713.46%, and the endogenous SNOs contents were enhanced by 74.65%; 93.49%; and 94.85%; by N6022 and GSNO treatments and *SlGSNOR* silencing, respectively. Altogether, these results indicate that SlGSNOR positively promotes tomato postharvest fruit ripening, which may be largely on account of its negative roles in the endogenous NO level.

## 1. Introduction

Fruit ripening is a highly complicated and irreversible phenomenon involving a series of physiological and biochemical changes correlated with fruit color, texture, flavor, and aroma [1]. Tomatoes are one of the most widely cultivated and consumed crops in the world, attributed to its rich antioxidants, vitamins, and bioactive phenolic compounds content. During tomato ripening, the skin color of the fruit undergoes a transition from green to red in most of the tomato varieties [2]. Usually, the color change comes along with the conversion of chloroplasts to chromoplasts; decrease of chlorophyll biosynthesis; degradation of chlorophylls; and accumulation of carotenoids and anthocyanins. It is known that the degradation of chlorophylls is conducted by chlorophyllase [3]. During chlorophyll biosynthesis, porphobilinogen deaminase (PBGD) functions in the early stage of chlorophyll biosynthesis; uroporphyrinogen III synthase (UROS) acts in the formation of uroporphyrinogen III, the precursor of all cellular tetrapyrroles including chlorophylls; and magnesium chelatase (MgCH) helps to insert Mg into the chlorophyll precursor [4]. Lycopene and β-carotene are principal carotenoids to supply visual cues of ripe fruit. The production of carotenoids is catalyzed by phytoene synthase (PSY); phytoene desaturase (PDS); ζ-carotene desaturase (ZDS); carotenoid isomerase (CRTISO); and lycopene β-cyclase (LYC) [3]. Between them, PSY catalyzes the first step in synthesizing carotenoids to product phytoene; phytoene desaturase (PDS) and ζ-carotene desaturase (ZDS) carry out the desaturation of phytoene and ζ-carotene to generate red-colored lycopene; and the red lycopene is converted to orange β-carotene through lycopene β-cyclase (LYC) [5]. In addition, tomato fruit softening occurs during ripening with a gradual decrease in firmness [6]. Softening is also one of the most significant factors in reducing the shelf life of tomatoes during postharvest storage. Fruit softening is caused by changes in the composition and structure of the cell wall, which is regulated by many softening-related enzymes, such as polygalacturonate (PG), pectate lyase (PL), pectin methyl esters (PME), and cellulase (Cx) [2]. In addition, there are many kinds of nutrients in ripe tomato fruit. At the onset of ripening, stored starch is metabolized to soluble sugars, which may also accelerate fruit softening [7]. Additionally, the concentration of acids, including ascorbic acid, citric acid, malic acid, etc., alters during fruit ripening. Phenols and flavonoids are also crucial nutrients in tomato fruit, which may influence the taste and flavor together with the sugars and acids [8]. Until now, multiple regulators have been found to mediate tomato fruit ripening. Furthermore, the research on tomato fruit concerning delaying postharvest senescence, inhibiting postharvest diseases, and sustaining wonderful nutritional quality during storage has been significant in recent years.

Nitric oxide (NO) is a small molecule gas that is widely distributed in plants. NO can freely pass through biological membranes, making NO an active signal within and between cells. NO participates in many physiological processes in plants, including cellular respiration; stomatal movement; seed germination; adventitious rooting; biotic and abiotic stress resistance; fruit ripening, and postharvest preservation [9,10]. As NO is unstable in the plant, it usually exists in the form of *S*-nitrosoglutathione (GSNO) [11]. Alternatively, the GSNO level is always in equilibrium with the protein *S*-nitrosothiols (SNOs), which may affect protein *S*-nitrosylation reactions [12]. In addition, GSNO can be irreversibly degraded by *S*-nitrosoglutathione reductase (GSNOR) [13,14]. GSNOR modulates the transnitrosylation equilibrium between GSNO and *S*-nitrosylated proteins, and, consequently, indirectly regulates cellular NO homeostasis [15]. Meanwhile, GSNOR is regarded as a housekeeping factor during plant growth and development, and abnormal GSNOR always triggers severe growth defects. For example, tomato *SlGSNOR*-overexpressed plants were stunted in plant growth with reduced seed germination and delayed flowering phenotypes [16]. Additionally, in tomato plants, SlGSNOR is widely involved in biological processes. For example, in axillary buds, the removal of bud dormancy was correlated with the inhibition of *SlGSNOR* [17]. Overexpressed *SlGSNOR* leads to enhanced resistance of tomato plants against *Alternaria solani*, via delaying symptom development and decreasing disease severity [18]. SlGSNOR also takes part in governing plant abiotic stress responses, photosynthesis, shoot side branching, and pollen and fruit development [19,20]. Recent studies have found that GSNOR participates in the ripening processes of many fruit, including sweet cherry [21], pepper [22], peach [23], and *Lotus japonicus* [24]. Moreover, GSNOR regulates fruit ripening through judging endogenous NO and protein *S*-nitrosylation levels, influencing phytohormone homeostasis, and cooperating with other ripeness regulators [23,25]. As in the tomato, overexpression or inhibition of *SlGSNOR* leads to abnormal fruit shape, and altered fruit weight and number [18,25]. However, the accurate mechanism concerning SlGSNOR, tomato fruit ripening, and postharvest fresh-keeping is unclear.

In this study, tomatoes (*Solanum lycopersicum* L. ‘Micro-Tom’) were used to detect the function of SlGSNOR in postharvest fruit ripening. In addition, the relationship between SlGSNOR and NO during tomato postharvest storage was discussed. In the present study, the GSNOR inhibitor N6022, NO donor GSNO, and a transgenic TRV-*SlGSNOR* system that could efficiently silence *SlGSNOR* and reduce the corresponding enzyme activity were employed. In addition, the content of chlorophyll, total carotenoid, and lycopene; the changes in color and firmness; and the accumulation of soluble sugar, soluble protein, titratable acid, ascorbic acid, total phenol, flavonoid, SNOs, and NO were determined in the treated tomato fruit. Our work offers new perspectives for the further study of postharvest fruit ripening.

## 2. Results

### 2.1. GSNOR Inhibitor and NO Donor Delayed Postharvest Tomato Fruit Ripening through Regulating Pigment Metabolisms

**Figure 1:**
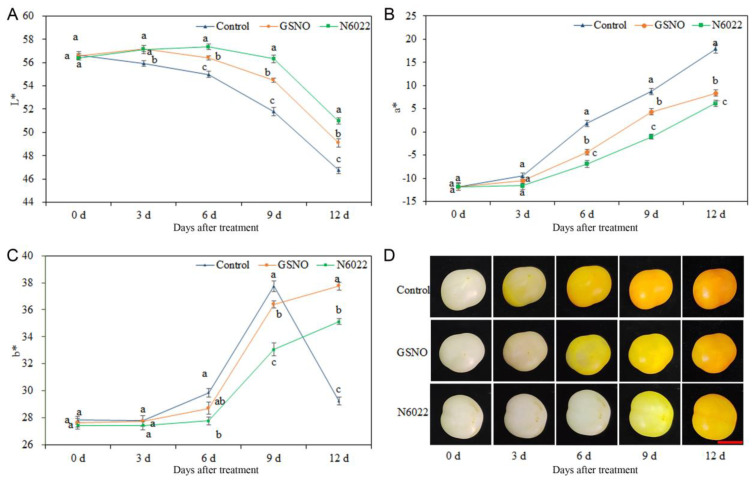
Effects of GSNO and N6022 on postharvest tomato fruit coloring. (**A**) L* value; (**B**) a* value; (**C**) b* value; (**D**) phenotype of tomato fruit in control, N6022, and GSNO treatments, days post treatment (bar = 1 cm). Different lowercase letters denote statistically significant distinctions within different treatments according to Duncan’s test (*p* < 0.05). L* indicates lightness; a* indicates range between green and red; and b* indicates range between blue and yellow.

To confirm the inhibitory effect of N6022 on GSNOR, we first detected the expression of *SlGSNOR* and its enzyme activity in N6022 treatment. As shown in Figure S1, the transcriptional level of *SlGSNOR* and SlGSNOR activity were significantly decreased by N6022. Thus, N6022 was applied as the GSNOR inhibitor in our study. To observe the change of skin color in tomato fruit, the values of L*, a*, and b* in tomato fruit in different treatments were measured. As shown in Figure 1, the L* values showed an overall decreasing trend with increasing storage time after treatment. The L* values under GSNO and N6022 treatments were higher than those in the control. In addition, the L* values under GSNO treatment were significantly lower than those under N6022 treatment from 6 to 12 d (Figure 1A). This indicated that the fruit skin lightness showed a gradual decrease with the increase in storage time after treatment, and the reduction of fruit skin lightness was delayed by GSNO and N6022 treatments. The a* values showed an upward trend with the increase in storage time after treatment. Additionally, the a* values in GSNO and N6022 treatments were all lower than those in the control during the study period. Compared with GSNO treatment, N6022 treatment lowered the a* values at each storage time point (Figure 1B). This revealed that the fruit skin color changed from green to red with the rise of storage time after treatment, and the reddening of fruit skin color was retarded by GSNO and N6022 treatments. The b* values showed an overall increasing trend with the increase in storage time after treatment. Notably, the b* value in the control was decreasing by day 12. In addition, the b* values in GSNO and N6022 treatments were lower than those in the control from day 6 to day 9. Meanwhile, the b* values in the N6022 treatment were lower than those in the GSNO treatment from day 6 to day 9. Interestingly, the b* values in the GSNO and N6022 treatments were significantly higher than those in the control on day 12 (Figure 1C). This suggested that the yellow color of the fruit skin firstly increased and then decreased with the increase in storage time after treatment, and the increase in the yellow color of the fruit skin was delayed by GSNO and N6022 treatments. As shown in Figure 1D, the skin color of the tomato fruit began to turn yellow on day 3 in the control. However, the skin color began to turn yellow on day 6 and day 9 in the GSNO and N6022 treatments, respectively.

**Figure 2:**
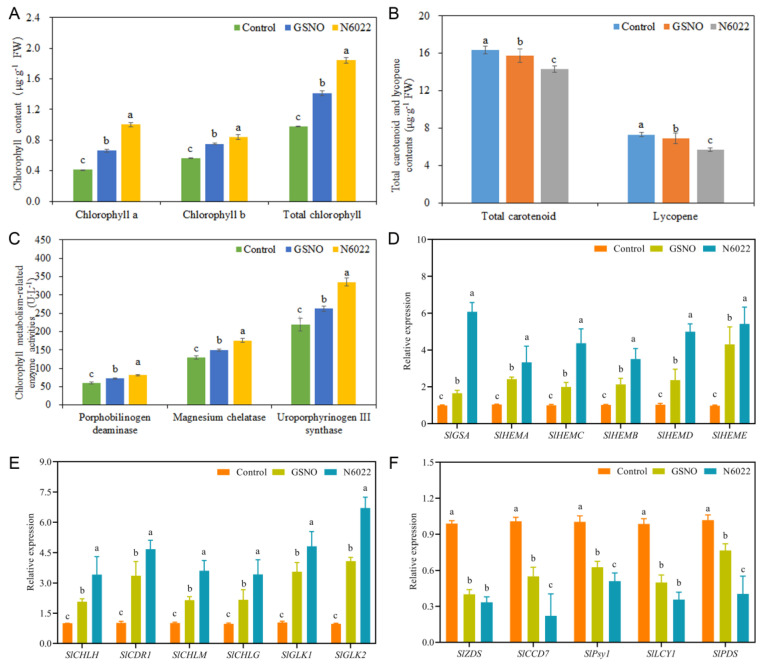
Effects of GSNO and N6022 on chlorophyll, total carotenoid, and lycopene contents, pigment metabolism-related enzyme activity, and related gene expressions. (**A**) Chlorophyll a, chlorophyll b, and total chlorophyll contents; (**B**) total carotenoid and lycopene contents; (**C**) chlorophyll metabolism-related enzyme activity; (**D**) expressions of *SlGSA*, *SlHEMA*, *SlHEMC*, *SlHEMB*, *SlHEMD*, and *SlHEME*; (**E**) expressions of *SlCHLH*, *SlCDR1*, *SlCHLM*, *SlCHLG*, *SlGLK1*, and *SlGLK2*; (**F**) Expressions of *SlZDS*, *SlCCD7*, *SlPsy1*, *SlLCY1*, and *SlPDS*. Error bars represent standard deviations (*n* = 3). Different lowercase letters denote statistically significant distinctions within different treatments according to Duncan’s test (*p* < 0.05).

Compared with the control, both the GSNO and N6022 treatments significantly increased the chlorophyll a, chlorophyll b, and total chlorophyll content by 59.60%; 33.83%; and 44.78%, and 141.53%; 49.38%; and 88.57%, respectively. Compared with the GSNO treatment, N6022 treatment significantly increased those contents (Figure 2A). However, both GSNO and N6022 treatments significantly reduced total carotenoid and lycopene content. N6022 treatment significantly reduced total carotenoid and lycopene content compared with the GSNO treatment (Figure 2B). As shown in Figure 2C, both GSNO and N6022 treatments significantly increased the activity of porphobilinogen deaminase, magnesium chelatase, and uroporphyrinogen III synthase compared with the control. Compared with the GSNO treatment, N6022 treatment significantly increased the activity of those enzymes.

In order to understand the metabolic mechanism of pigments in tomato fruit ripening, the transcript abundances of several pigment-related genes were determined (Figure 2). Compared with the control, both GSNO and N6022 treatments significantly increased the transcript abundances of *SlGSA*, *SlHEMA*, *SlHEMC*, *SlHEMB*, *SlHEMD*, *SlHEME*, *SlCHLH*, *SlCDR1*, *SlCHLM*, *SlCHLG*, *SlGLK1*, and *SlGLK2*. In addition, compared with the GSNO treatment, N6022 treatment strengthened the transcript abundances of those genes by 267.85%, 37.54%, 119.55%, 64.84%, 111.58%, 25.91%, 85.11%, 65.70%, 39.41%, 68.22%, 53.00%, 58.57%, 35.21%, and 64.38%, respectively (Figure 2D,E). Interestingly, compared with the control, treatments with GSNO and N6022 observably reduced the expression levels of *SlZDS, SlCCD7, SlPsy1, SlLCY1, and SlPDS*. Meanwhile, the expression levels of *SlCCD7, SlPsy1, and SlPDS* were significantly reduced by N6022 treatment compared with the GSNO treatment. However, there was no obvious difference in the expression levels of *SlZDS* and *SlLCY1* between GSNO and N6022 treatments (Figure 2F).

### 2.2. GSNOR Inhibitor- and NO Donor-Delayed Postharvest Tomato Fruit Ripening through Regulating Fruit Firmness

**Figure 3:**
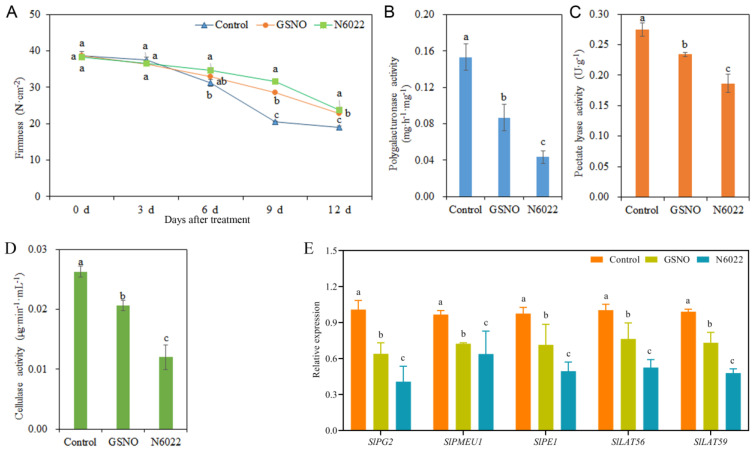
Effects of GSNO and N6022 on tomato fruit firmness, enzyme activity of polygalacturonase; pectate lyase; and cellulase, and related gene expressions. (**A**) Firmness; (**B**) polygalacturonase activity; (**C**) pectate lyase activity; (**D**) cellulase activity; (**E**) expressions of *SlPG2*, *SlPMEU1, SlPE1*, *SlLAT56*, and *SlLAT59*. Error bars represent standard deviations (*n* = 3). Different lowercase letters denote statistically significant distinctions within different treatments according to Duncan’s test (*p* < 0.05).

To explore the effect of the GSNOR inhibitor and NO donor on the softening of postharvest tomato fruit, the fruit firmness; the activity of firmness-related enzymes; and the transcript levels of fruit firmness-related genes were examined (Figure 3). The fruit firmness showed a downward trend with the increase in storage time after treatment. Interestingly, fruit firmness was significantly higher in the GSNO and N6022 treatments than in the control from day 9 to day 12. Meanwhile, fruit firmness in the N6022 treatment was significantly higher than that in the GSNO treatment from day 9 to day 12. Fruit firmness was higher in the N6022 treatment than in the control on day 6 (Figure 3A). Compared with the control, both GSNO and N6022 treatments significantly reduced the activity of polygalacturonase, pectate lyase, and cellulase enzyme. Meanwhile, compared with the GSNO treatment, N6022 treatment observably reduced the activity of those enzymes (Figure 3B–D). Compared with the control, both GSNO and N6022 treatments significantly reduced the transcript abundances of *SlPG2, SlPMEU1, SlPE1*, *SlLAT56*, and *SlLAT59*. Additionally, compared with the GSNO treatment, N6022 treatment observably reduced the transcript abundances of those genes (Figure 3E).

### 2.3. GSNOR Inhibitor- and NO Donor-Altered Nutrient Contents in Postharvest Tomato Fruit

**Figure 4:**
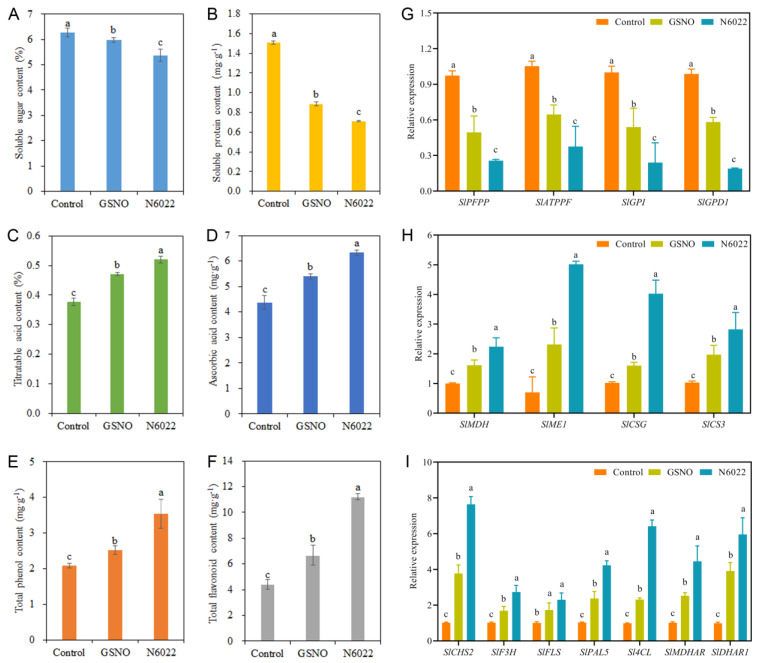
Effects of GSNO and N6022 on soluble sugar; soluble protein; titratable acid; ascorbic acid; total phenol; and total flavonoid contents, and nutrient-related gene expressions in postharvest tomato fruit. (**A**) Soluble sugar content; (**B**) soluble protein content; (**C**) titratable acid content; (**D**) ascorbic acid content; (**E**) total phenol content; and (**F**) total flavonoid content. (**G**) Expressions of *SlPFPP*, *SlATPPF*, *SlGPI*, and *SlGPD1*; (**H**) expressions of *SlMDH*, *SlME1*, *SlCSG*, and *SlCS3*; and (**I**) expressions of *SlCHS2*, *SlF3H*, *SlFLS*, *SlPAL5*, *Sl4CL*, *SlMDHAR*, and *SlDHAR1*. Error bars represent standard deviations (*n* = 3). Different lowercase letters denote statistically significant distinctions within different treatments according to Duncan’s test (*p* < 0.05).

To investigate the effects of GSNOR inhibitor and NO donor on postharvest fruit nutrient content, the contents of soluble sugar, soluble protein, titratable acid, ascorbic acid, total phenol, and total flavonoid were measured (Figure 4). Both GSNO and N6022 treatments significantly reduced the contents of soluble sugar and protein compared with the control. Compared with GSNO treatment, the N6022 treatment reduced soluble sugar and protein contents (Figure 4A,B). However, both GSNO and N6022 treatments significantly increased the contents of titratable acid, ascorbic acid, total phenol, and total flavonoid compared with the control. Those contents were observably strengthened by the N6022 treatment compared with the GSNO treatment (Figure 4C–F).

Compared with the control, both GSNO and N6022 treatments significantly reduced the transcript abundances of *SlPFPP*, *SlATPPF*, *SlGPI*, and *SlGPD1* (Figure 4). Compared with the control, GSNO treatment reduced the expression of these genes by 49.13%, 38.62%, 46.11%, and 41.05%, respectively. In addition, compared with the GSNO treatment, N6022 treatment signally reduced the transcript levels of these genes (Figure 4G). Compared with the control, both GSNO and N6022 treatments observably strengthened the transcript abundances of *SlMDH*, *SlME1*, *SlCSG*, *SlCS3*, *SlCHS2*, *SlF3H*, *SlFLS*, *SlPAL5*, *Sl4CL*, *SlMDHAR*, and *SlDHAR1*, and the expressions of these genes in GSNO treatment were strengthened by 62.06%, 231.07%, 57.25%, 91.99%, 273.09%, 65.50%, 70.92%, 132.11%, 134.79%, 148.54, and 298.19%, respectively. Furthermore, compared with GSNO treatment, the expression levels of these genes (except *SlFLS*) were significantly increased by N6022 treatment (Figure 4H,I).

### 2.4. GSNOR Inhibitor- and NO Donor-Enhanced Endogenous NO Content in Postharvest Tomato Fruit

**Figure 5:**
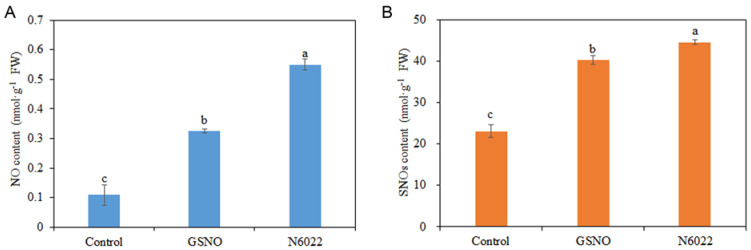
Effects of GSNO and N6022 on NO and SNOs contents. (**A**) NO content; (**B**) SNOs content. Error bars represent standard deviations (*n* = 3). Different lowercase letters denote statistically significant distinctions within different treatments according to Duncan’s test (*p* < 0.05).

As shown in Figure 5, compared with the control, NO content was significantly increased by 404.59% and 197.55%, and SNOs content was enhanced by 93.49% and 74.65% by GSNO and N6022 treatments, respectively. In addition, N6022 treatment significantly increased the contents of NO and SNOs compared with GSNO treatment (Figure 5).

### 2.5. Silencing of SlGSNOR-Enhanced NO Content in Postharvest Tomato Fruit

**Figure 6:**
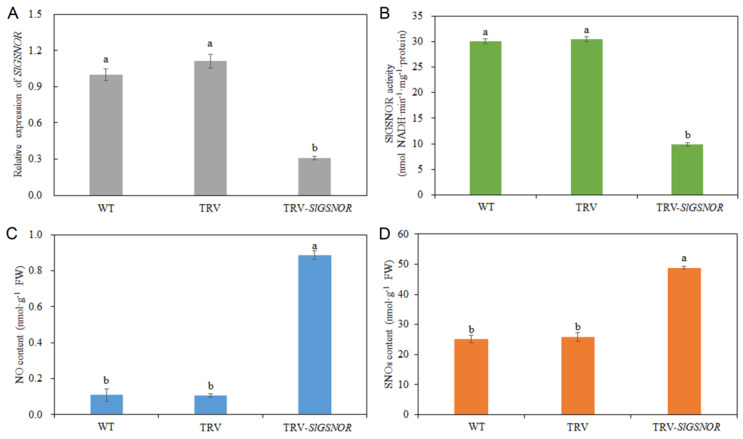
Effects of *SlGSNOR* silencing on *SlGSNOR* gene expression, SlGSNOR enzyme activity, NO content, and SNOs content. (**A**) Expression of *SlGSNOR*; (**B**) SlGSNOR activity; (**C**) NO content; (**D**) SNOs content. Error bars represent standard deviations (*n* = 3). Different lowercase letters denote statistically significant distinctions within different treatments according to Duncan’s test (*p* < 0.05).

Compared with wild-type (WT) and empty TRV (pTRV1 + pTRV2), TRV-*SlGSNOR* significantly decreased the expression of *SlGSNOR* (Figure 6A). Similarly, TRV-*SlGSNOR* markedly repressed SlGSNOR activity compared to WT and empty TRV (Figure 6B). On the contrary, the NO content in TRV-*SlGSNOR*-infected fruit was significantly higher than that in WT- and empty TRV-infected fruit, by 713.46% and 733.89%, respectively (Figure 6C). Meanwhile, compared to WT and empty TRV, TRV-*SlGSNOR* markedly enhanced SNOs content by 94.85% and 89.08%, respectively (Figure 6D).

### 2.6. Silencing of SlGSNOR-Suppressed Postharvest Tomato Fruit Ripening

**Figure 7:**
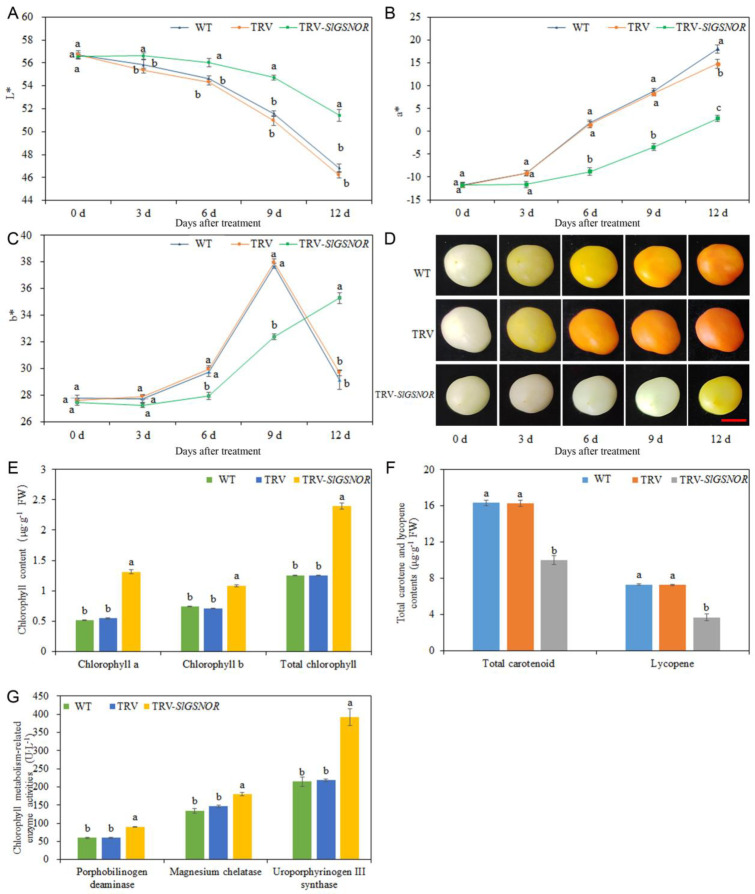
Effect of *SlGSNOR* silencing on postharvest tomato fruit coloring. (**A**) L* value; (**B**) a* value; (**C**) b* value; (**D**) phenotype of tomato fruit in wild-type (WT), TRV, and TRV-*SlGSNOR*, days post treatments (bar =1 cm); (**E**) chlorophyll a, chlorophyll b, and total chlorophyll content; (**F**) total carotenoid and lycopene content; and (**G**) chlorophyll metabolism-related enzyme activity. Error bars represent standard deviations (*n* = 3). Different lowercase letters denote statistically significant distinctions within different treatments according to Duncan’s test (*p* < 0.05). L* indicates lightness; a* indicates range between green and red; and b* indicates range between blue and yellow.

A gradual decrease in L* values was observed as the number of days increased. The L* values in TRV-*SlGSNOR*-infected fruit were significantly higher than those in WT- and empty TRV-infected fruit at 3, 6, 9, and 12 d, which indicated a delayed decrease in fruit skin lightness in SlGSNOR-silencing fruit (Figure 7A). However, the a* value showed the opposite trend to the L* value. Compared with WT- and empty TRV-infected fruit, TRV-*SlGSNOR*-infected fruit significantly decreased the a* values between 6 and 9 d, which revealed a delayed increase in fruit skin color changing from green to red in SlGSNOR-silencing fruit (Figure 7B). The b* values in WT- and empty TRV-infected fruit exhibited an increasing trend from 6 to 9 d and showed a decreasing trend in the time following, whereas TRV-*SlGSNOR*-infected fruit showed an increasing trend from 6 to 12 d. The b* values in TRV-*SlGSNOR*-infected fruit were significantly lower than those in WT- and empty TRV-infected fruit from 6 to 9 d, while showing the opposite trend from 9 to 12 d, which suggested a delayed increase in fruit skin yellow color in *SlGSNOR*-silencing fruit (Figure 7C). Meanwhile, compared with WT and empty TRV, TRV-*SlGSNOR* delayed the changes in skin color (Figure 7D). Compared to those in WT- and empty TRV-infected fruit, the chlorophyll a, chlorophyll b, and total chlorophyll content in TRV-*SlGSNOR*-infected fruit was increased by 154.36%; 46.85%; and 91.03%, and 139.92; 53.79%; and 91.43%, respectively (Figure 7E). Moreover, the total carotenoid and lycopene content in TRV-*SlGSNOR*-infected fruit was significantly lower than those in WT- and empty TRV-infected fruit (Figure 7F). The chlorophyll metabolism-related enzyme activity in TRV-*SlGSNOR*-infected fruit was significantly higher than those in WT- and empty TRV-infected fruit (Figure 7G).

As shown in Figure S2A, the expression levels of *SlGSA*, *SlHEMA*, *SlHEMC*, *SlHEMB*, *SlHEMD*, and *SlHEME*, in TRV-*SlGSNOR*-infected fruit were significantly higher than those in WT- and empty TRV-infected fruit. Meanwhile, compared with WT- and empty TRV-infected fruit, the expression levels of *SlCHLH*, *SlCDR1*, *SlCHLM*, *SlCHLG*, *SlGLK1*, and *SlGLK2* were enhanced in TRV-*SlGSNOR*-infected fruit (Figure S2B). In addition, TRV-*SlGSNOR* distinctly decreased the expression levels of *SlZDS*, *SlCCD7*, *SlPsy1*, *SlLCY1*, and *SlPDS* compared to WT and empty TRV (Figure S2C).

**Figure 8:**
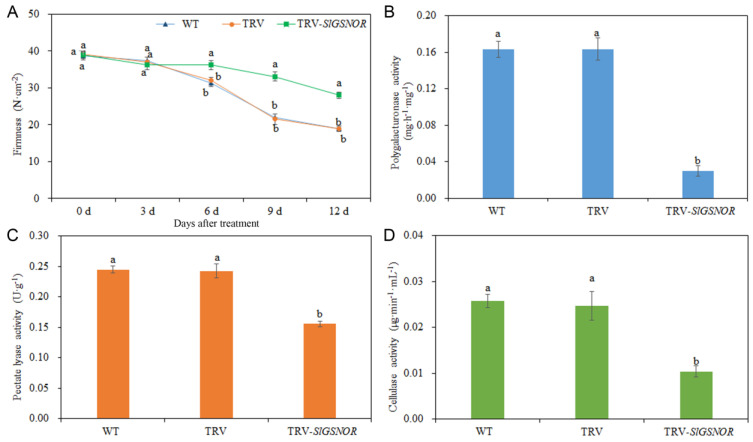
Effect of *SlGSNOR* silencing on postharvest tomato fruit softening. (**A**) Firmness; (**B**) polygalacturonase activity; (**C**) pectate lyase activity; (**D**) cellulase activity. Error bars represent standard deviations (*n* = 3). Different lowercase letters denote statistically significant distinctions within different treatments according to Duncan’s test (*p* < 0.05).

As shown in Figure 8A, compared with WT and empty TRV, TRV-*SlGSNOR* significantly enhanced firmness between 6 and 12 d. There were no significant differences in fruit firmness between WT- and TRV-*SlGSNOR*-infected fruit from 0 to 3 d. Additionally, compared to WT- and empty TRV-infected fruit, the polygalacturonase activity in TRV-*SlGSNOR*-infected fruit was reduced (Figure 8B). TRV-*SlGSNOR* markedly decreased the pectate lyase activity compared with WT and empty TRV (Figure 8C). Meanwhile, in comparison with WT and empty TRV, TRV-*SlGSNOR* also significantly repressed cellulase activity (Figure 8D).

As shown in Figure S3, the expression levels of *SlPG2, SlPMEU1*, *SlPE1*, *SlLAT56*, and *SlLAT59* in TRV-*SlGSNOR*-infected fruit were significantly lower than those in WT and empty TRV-infected fruit.

### 2.7. Silencing of SlGSNOR-Influenced Different Nutrient Contents in Postharvest Tomato Fruit

**Figure 9:**
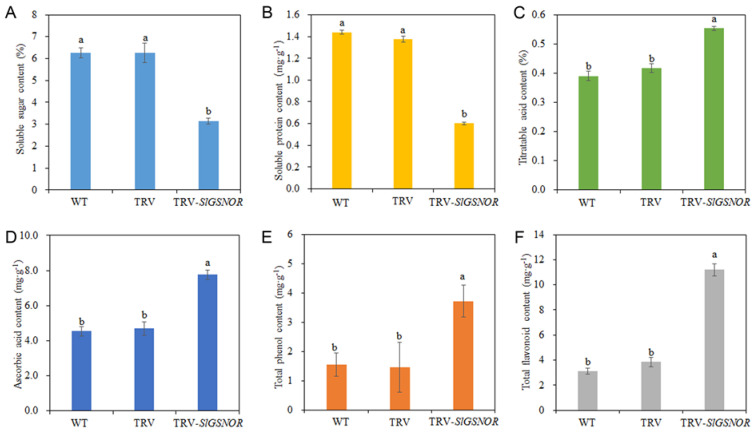
Effect of *SlGSNOR* silencing on different nutrient contents in postharvest tomato fruit. (**A**) Soluble sugar content; (**B**) soluble protein content; (**C**) titratable acid content; (**D**) ascorbic acid content; (**E**) total phenol content; (**F**) total flavonoid content. Error bars represent standard deviations (*n* = 3). Different lowercase letters denote statistically significant distinctions within different treatments according to Duncan’s test (*p* < 0.05).

Compared with WT and empty TRV, TRV-*SlGSNOR* significantly reduced soluble sugar content (Figure 9A). The soluble protein content in TRV-*SlGSNOR*-infected fruit was distinctly lower than WT- and empty TRV-infected fruit (Figure 9B). On the contrary, compared to WT and empty TRV, TRV-*SlGSNOR* significantly increased the titratable acid and ascorbic acid content (Figure 9C, D). Meanwhile, in comparison with WT and empty TRV, TRV-*SlGSNOR* remarkably elevated the total phenol content (Figure 9E). Similarly, the total flavonoid content in TRV-*SlGSNOR*-infected fruit was significantly higher than that in WT- and empty TRV-infected fruit (Figure 9F).

As shown in Figure S4A, compared with WT and empty TRV, TRV-*SlGSNOR* significantly reduced the expression levels of *SlPFPP*, *SlATPPF*, *SlGPI*, and *SlGPD1*. Also, the expression levels of *SlMDH*, *SlME1*, *SlCSG*, and *SlCS3* in TRV-*SlGSNOR*-infected fruit were significantly higher than those in WT- and empty TRV-infected fruit (Figure S4B). On the contrary, in comparison with WT and empty TRV, TRV-*SlGSNOR* significantly upregulated the expression levels of *SlCHS2*, *SlF3H*, *SlFLS*, *SlPAL5*, *Sl4CL*, *SlMDHAR*, and *SlDHAR1* (Figure S4C).

## 3. Discussion

The postharvest storage of tomato fruit is accompanied by multiple physiological and biochemical processes concerning fruit coloring, softening, and changes of nutritional quality. Recently, many regulators which participate in fruit ripening have been uncovered, including a GSNOR-directed NO signal. In pepper (*Capsicum annuum* L.), the process of fruit ripening is accompanied by progressively weakened GSNOR activity [22]. In tomato, *SlGSNOR* is highly expressed in fruit during ripening [26]. Additionally, the depletion or overexpression of *SlGSNOR* may cause deficient fruit development phenotypes, such as abnormal fruit shape, and decreases in fruit size and fruit number [11,18]. Thus, the proper function of GSNOR is indispensable during fruit development. In this study, GSNOR inhibitor N6022 and a TRV-*SlGSNOR* transgenic system were used to restrain *SlGSNOR* expression and the corresponding enzyme activity in postharvest tomato fruit (Figure 6 and Figure S1). We found that the transition of tomato fruit skin color to yellow was delayed in SlGSNOR-defective plants, which was similar to that under the NO donor GSNO treatment (Figure 1 and Figure 7). As a kind of fleshy fruit, the change in skin color is one of the most important developmental acts correlated with ripening [3]. Thus, coloration is a crucial step during fruit ripening and fruit postharvest storage. The transition of tomato fruit skin color is always conducted via the destruction of chlorophylls and the accumulations of new pigments, such as carotenoid, lycopene, and anthocyanins [27]. NO can postpone fruit coloration through judging pigment biosynthesis. In *Lycium barbarum*, the application of NO donor sodium nitroprusside (SNP) repressed the accumulation of anthocyanins and increased the production of a colorless metabolite proanthocyanin, resulting in delayed fruit coloration [28]. In our study, the content of total chlorophyll was enhanced by 88.57%, 91.03%, and 44.78% by N6022, TRV-*SlGSNOR*, and GSNO treatments, respectively (Figure 2 and Figure 7). In addition, the levels of total carotenoid and lycopene were reduced by the above treatments during the postharvest stage (Figure 2 and Figure 7). Furthermore, the activity of chlorophyll biosynthesis enzymes and the expression levels of chlorophyll biosynthesis genes were upregulated, and the transcripts of carotenoid biosynthesis genes were downregulated by N6022, TRV-*SlGSNOR*, and GSNO (Figure 2). Similarly, the transcription of *phytoene synthase 2* (*PSY2*) and the bioproduction of carotenoid were restrained in tomato virus-induced *gsnor* mutant seedlings [29]. In addition, another study announced that exogenous NO decreased total carotenoid and lycopene levels during tomato fruit ripening [30]. Hence, the deficiency of GSNOR and the addition of NO may take part in postponing colored new pigment biosynthesis during postharvest storage. Meanwhile, NO seems to play a dual role in chlorophyll biosynthesis. In Chinese flowering cabbage, NO induced chlorophyll biosynthesis and dropped chlorophyll catabolism to relieve postharvest yellowing [31]. However, gene silencing of *GSNOR* triggered high levels of NO, which further inhibited the expression of *protochlorophyllide oxidoreductase C* (*PORC*), a rate-limiting enzyme in chlorophyll biosynthesis, and reduced chlorophyll accumulation [29]. Thus, the chlorophyll content may be differentially regulated by GSNOR and NO within different plant tissues during the postharvest stage, and the mechanism is waiting to be investigated further.

During fleshy fruit ripening, the pectin in the cell wall of fruit tissues is decomposed gradually, resulting in cell wall loosening and softening [32]. Previous studies have found that NO may alleviate postharvest fruit softening by mediating many cell wall softening-related factors. In papaya (*Carica papaya* L. cv ‘Sui you 2′), firmness was increased and the ethylene accumulation was reduced in preservable fruit fumigated with NO [2]. In addition, activity of the cell wall softening-related enzymes polygalacturonate (PG); pectate lyase (PL); pectin methyl esters (PME); and cellulase (Cx) were retarded by NO. In banana, the NO donor SNP delayed fruit slice softening through sustaining acid-soluble pectin and starch contents and reduced PG, PME, and endo-β-1,4-glucanase activity [33]. Similarly, SNP retained fruit mass, color, and firmness, and decreased PG, PME, and Cx activity during pear fruit storage [6]. In our study, the defective SlGSNOR and the addition of NO exhibited a deferred decrease of firmness, and lower activity of PG, PL, and Cx with the inhibited expressions of the related genes compared with the control (Figure 3 and Figure 8). As the function of GSNOR in fruit softening is unknown, our results suggest that GSNOR may serve as a positive regulator of postharvest fruit softening to maintain the integrity of cell wall structure, which is opposite to NO. Nevertheless, peach fruit treated with NO exhibited a higher value of firmness and a higher activity of GSNOR enzyme during early cold storage [34]. Thus, the influence of NO via GSNOR on the postharvest softening may be complicated within different postharvest stages and different plant species.

As the influence of GSNOR and NO on tomato postharvest fruit ripening is described, we further analyze the effect of these regulators on tomato postharvest nutrition. Upon ripening, the nutritious chemicals change, making the fruit delicious and attractive to animals in order to disperse mature seeds [35]. Sugars, acids, and volatiles are the main chemicals to fix the taste and flavor quality of tomato fruit [36]. As for GSNOR, the activity of GSNOR and DHAR were stimulated concurrently with the increase in ascorbic acid content in the antimony-treated roots of sunflower (*Helianthus annuus* L.) [37]. In tomato, the endogenous indole-3-acetic acid (IAA) content was obviously decreased in leaf primordium and early development fruit of *SlGSNOR*-deficient lines [25]. Additionally, both the GSNOR activity and the ascorbate level were reduced in tomato roots under salt stress [38]. However, the direct regulatory mechanism of GSNOR on acid biosynthesis is unclear. In recent years, some studies have uncovered the role of NO in sugar and acid accumulation in postharvest horticultural products. For instance, exogenous NO treatment triggered the accumulation of titratable acidity and ascorbic acid in postharvest citrus fruit [39]. Sweet cherry fruit immersed in NO-releasing chitosan nanoparticles exhibited a higher fruit weight, and a higher soluble solid and ascorbic acid content during postharvest storage [21]. In addition, the enzyme activity of DHAR and MDHAR concerning ascorbic acid metabolism were raised. Exogenous NO has also been found to improve ascorbic acid content in fresh-cut potatoes and tomato fruit [30,40]. Similarly, in our study, N6022 and GSNO facilitated the production of titratable acid and ascorbic acid, and up-regulated the transcripts of *SlMDHAR* and *SlDHAR1* in postharvest tomato fruit (Figure 4). As silencing of *SlGSNOR* introduced similar nutritional phenotypes as N6022 (Figure 9), we suggest that the deletion of GSNOR and the application of NO may elevate fruit acid content during storage. However, the effects of GSNOR and NO on soluble sugar and soluble protein accumulation are different. A previous study found that NO alleviated postharvest senescence in *Consolida ajacis* cut flowers by improving the levels of sugars, phenols and soluble proteins [41]. Nevertheless, another report found that the bioproduction of sugars, organic acids, and amino acids was mildly influenced by exogenous NO and indirectly stimulated GSNOR during tomato storage [30]. In our study, N6022, TRV-*SlGSNOR*, and GSNO synchronously repressed the accumulation of soluble sugar and soluble protein, and reduced the expression levels of sugar metabolism-related genes (Figure 4, Figure 9 and Figure S4). Thus, our results suggest that the SlGSNOR and NO inversely regulated the biosynthesis of sugars and acids in postharvest tomato fruit. As an increase in soluble sugar may promote fruit softening [7], the retarded accumulation of soluble sugar may be one of the reasons for the repression of postharvest fruit softening by the defective SlGSNOR. Meanwhile, the impacts of GSNOR and NO on these nutrients may be different upon different treatment conditions and different storage periods. It has been found that phenolic acids and flavonoids are essential compounds for sustaining great quality in postharvest fruit [27]. Also, they are a crucial source for tomato flavor construction [42]. In *Inonotus obliquus*, both higher cellular NO content and GSNOR inhibition could raise polyphenol production, illustrating the role of NO and GSNOR in phenol biosynthesis [43]. In NO-treated mango fruit, the amount of total phenol and total flavonoid was promoted, which contributed to restricting pathogen growth during storage [44]. In the storage of fresh-cut potatoes, NO donor SNP treatment increased the levels of total phenol and total flavonoid to maintain flavor quality [40]. Being similar, in our study, the contents of total phenol and total flavonoid were upregulated by the inhibition of SlGSNOR and the appending of NO (Figure 4 and Figure 9). Thus, GSNOR and NO may take part in regulating total phenol and flavonoid accumulations in postharvest fruit.

Previous studies have announced that GSNOR may participate in mediating plant growth and development through maintaining endogenous NO and SNOs homeostasis [11,25]. In addition, the inhibition of GSNOR always comes along with the increase in endogenous NO and SNOs content and the level of protein *S*-nitrosylation [45]. For example, the application of gamma-aminobutyric acid helped retain NO equilibrium in harvested tomatoes to resist *Botrytis cinerea* infection via mediating GSNOR [46]. In *SlGSNOR* knockdown tomato plants, the NO content in roots and fruit, and the SNOs content in leaves were elevated [20,25]. In our results, the inhibition of SlGSNOR by N6022 and TRV-*SlGSNOR* infection within wild-type tomato fruit also induced improved accumulation of NO and SNOs during storage (Figure 5 and Figure 6). Thus, GSNOR may influence postharvest physiological processes through judging endogenous NO and SNOs levels. In addition, peach fruit treated with N6022 exhibited a higher content of NO and SNOs than that treated with exogenous NO [23]. Similarly, N6022 triggered a stronger effect on NO and SNOs accumulation than GSNO treatment in our results. As SNOs serves as natural reservoirs of NO [15], our results and previous studies suggest that the deficiency of GSNOR in plants may effectively enhance endogenous NO content. Also, the accumulation of NO by GSNOR may be an important mechanism in GSNOR-directed postharvest fruit ripening; however, the precise mechanism needs to be studied further.

## 4. Materials and Methods

### 4.1. Plant Materials and Treatments

Tomato (*S. lycopersicum* cv. Micro-Tom) fruit were used as plant materials, and the corresponding tomato seeds were reproduced and stored in our own lab. Firstly, tomato seeds with grains and consistent size were selected and sterilized with 1% NaClO solution for 15 min. Secondly, these seeds were put into a conical flask with 50 mL distilled water, and the flask was placed in a HYG-C type shaker and cultured at a rotation speed of 200 r min−1 at 26 ◦C for 3 d to facilitate germination. Thirdly, the germinated seeds were collected and transferred into distilled water for 7 d. Then, the seedlings were transferred into a Hogland solution for growth, and the Hogland solution was replaced once a week. Tomato plants were grown in a light incubator. The condition of the incubator was 16 h light at 18,000 Lx at 26 ± 2 °C, and 8 h dark at 18 ± 2 °C; the relative humidity was kept at 85%. The healthy and uniform tomato fruit were harvested 30 days after pollination (DPA). These fruit were injected only once with 150 µL of N6022 (60 µM) [23]; GSNO (100 µM, which was obtained from our preliminary experiments); and sterile water (control). Then, these treated fruit were placed into the incubator and incubated in the dark. During storage, the treated samples were harvested on 0 d, 3 d, 6 d, 9 d, and 12 d, separately. There were 3 biological replicates with each replicate composed of the pericarp from at least 6 fruit. The pericarp of the samples harvested at each time point was used for the detection of chromatic aberration and the firmness of fruit. Meanwhile, the pericarp of each sample treated for 9 d was used to measure the physiological and molecular phenotypes.

### 4.2. Virus-Induced Gene Silencing (VIGS) of SlGSNOR

The tobacco rattle virus (TRV)-based vectors pTRV1 and pTRV2 were utilized in the present study [47]. A fragment of *SlGSNOR* within the *SlGSNOR* gene coding sequence was cloned into the pTRV2 vector to generate the pTRV2-*SlGSNOR* recombinant construction. VIGS was conducted via infiltration into 30 DPA tomato fruit with a mixture of *Agrobacterium* tumefaciens carrying pTRV1 and pTRV2-*SlGSNOR* plasmid, as described previously [47]. The primers used to amplify the *SlGSNOR* fragment are listed in Table S1. The fruit infected with the empty pTRV2, and WT fruit without any infection were used as controls. The injection was administered once, and samples were harvested on 0 d, 3 d, 6 d, 9 d, and 12 d after injection, respectively.

### 4.3. Detection of SlGSNOR Enzyme Activity

The method of Zuccarelli et al. (2017) was used to determine SlGSNOR enzyme activity with some modifications [48]. The gathered fruit pericarps were pulverized to powder in a mortar in liquid nitrogen. Then, 1 g of the powder was put into 2 mL of reaction solution (20 mM Tris-HCl, pH 8.0, 0.2 mM NADH, 0.5 mM EDTA) and mixed thoroughly. After centrifugation at 10,000× *g* for 20 min, the collected supernatant was mixed with 100 μL of 4 mM GSNO. After incubation for 30 min at 4 °C, the absorbance of the supernatant was measured at 340 nm using a spectrophotometer to determine the activity of SlGSNOR via changes of NADH decomposition (UV-1800, Shimadzu, Japan). The SlGSNOR activity was demonstrated as 1 mg of protein consuming nM NADH in 1 min (є = 6.22 nm^−1^ cm^−1^).

### 4.4. Determination of SNOs and NO Contents

The Saville–Gress method was used to determine the SNOs content with minor revisions [49]. After grinding 1 g of each sample into powder with liquid nitrogen, 3 mL of extraction buffer (50 mM Tris-HCl, pH 8.0, 150 mM NaCl) containing 1 mM protease inhibitor (PMSF) was added into the ground samples, and incubated on ice for 20 min. After centrifugation (10,000× *g*, 15 min, 4 °C), 250 μL of supernatant was mixed with 250 μL of 1% sulfanilamide [with or without 0.2% (*w*/*v*) HgCl_2_], and incubated in darkness on ice for 20 min. Then, 100 μL of 0.02% NED [N-(1-naphthyl) ethylenediamine] was added, and the mixture was incubated on ice for 20 min. An enzyme standard instrument (CMax Plus, CA, USA) was used to measure the absorbance at 540 nm.

The content of NO was measured via the Greiss reagent method as described in Zhu et al. (2016) [50]. The samples were pulverized to powder in a mortar with liquid nitrogen. Then, 0.5 g of the powder was put into 1 mL of extraction solution (50 mM glacial acetic acid + 4% zinc diacetate) and mixed thoroughly. After centrifugation (10,000× g, 15 min, 4 °C), the collected supernatant was mixed with 0.1 g of activated carbon. After filtration, 0.4 mL of the reaction solution was mixed with 0.4 mL of Greiss reagent (1% p-aminobenzene sulphonic acid, 0.1% N-naphthalene ethylenediamine, and 5% phosphoric acid). After incubation for 30 min at 25 °C, the absorbance of the supernatant was measured at 550 nm using a spectrophotometer (UV-1800, Shimadzu, Japan).

### 4.5. Measurement of Fruit Chromatic Aberration and Firmness

The fruit were photographed at each sampling time point with a camera (750D, Canon, Tokyo, Japan). The skin color of the fruit was measured with a colorimeter (CR-10 Plus, Konica Minolta, Inc., Tokyo, Japan). The 3 values L*, a* and b* on the colorimeter surface represent different color surface coordinates. In detail, the values of L*, a*, and b* indicate lightness, a range between green and red, and a range between blue and yellow, respectively. Each sample was randomly selected, and 3 measurements were performed on the top of each fruit, at a point parallel to the equatorial plane, and at the shoulder, respectively. The fruit firmness was determined via a hand-held digital fruit firmness tester (GY-4-J, Top Cloud-agri Technology Co., Ltd., Hangzhou, China).

### 4.6. Measurement of Nutritional Phenotypes

The soluble sugar, soluble protein, ascorbic acid, total phenol, and total flavonoid contents were measured as previously described in Zhang et al. (2023) [51]. The titratable acid content was determined according to the method of Adhikary et al. (2021) with slight modifications [1]. In the present study, 5 g of fresh sample and 2 g of quartz sand were added into a mortar, and the mixture was thoroughly ground with a pestle to homogenate. The homogenate was collected and mixed thoroughly with 30 mL of distilled water in an Erlenmeyer flask. Then, the Erlenmeyer flask was placed into a laboratory water bath, and incubated at 80 °C for 30 min. The extract was filtered with a chemical analysis filter paper into a new conical flask, and the extracted liquid was added with distilled water to 10 mL. Then, 2 drops of phenolphthalein indicator were added in the conical flask. Titratable acid was measured by titrating the solution with sodium hydroxide (0.1 mol L^−1^) to pH 8.3, and the content was expressed as a percentage of the volume added to the total volume of the final solution.

### 4.7. Detection of Fruit Pigment Contents

The frozen fruit pericarp was pulverized to powder in a mortar in liquid nitrogen. Then, 0.5 g of the powder was put into 3 mL of 80% acetone, and placed in darkness for 5 min. The extracted liquids were filtered with a chemical analysis filter paper, and the collected supernatant was used for analyzing chlorophyll b, chlorophyll a, and total chlorophyll contents at 445.5 nm, 649 nm, and 665 nm, with a spectrophotometer (UV-1800, Shimadzu, Japan), respectively. The method in El-Mergawi et al. (2014) was used to determine total carotenoid and lycopene content with some modifications [52]. The sample was ground to powder with liquid nitrogen. Then, 2 g of the powder was extracted with acetone/ethanol (1:1, *v*/*v*). After centrifugation (1500× g, 15 min, 4 °C), the supernatant was collected and used to determine the absorbance values at 470 nm with a spectrophotometer (UV-1800, Shimadzu, Japan). The particular extinction coefficient (E 1% 1 cm) of 2500 was used to compute the total carotenoid content. As for lycopene content detection, a solution composed of hexane, acetone, and ethanol (2:1:1, *v*:*v*:*v*) was added into 2 g of the above powder. The supernatant was used for absorbance measurement at 472 nm with a spectrophotometer (UV-1800, Shimadzu, Japan), and the hexane solution was used as a blank control. The lycopene’s specific extinction coefficient (E 1% 1 cm) of 3450 in hexane was used to determine the lycopene content.

### 4.8. Determination of Chlorophyll and Nutrition-Related Enzyme Activity

The activity of PBGD, MgCH, and UROS were determined via an ELISA kit, as described in Jiao et al. (2017) [53]. The measurements of PG, PL, and Cx activity were based on the method described by Guo et al. (2014) [2]. In our study, 1 g of fresh fruit pericarp in each sample was used in the measurement of enzyme activity. All the experimental steps were performed at 4 °C. A spectrophotometer (UV-1800, Shimadzu, Japan) was applied to detect the absorbances, with the wave lengths of PBGD, MgCH, UROS, PG, PL, and Cx at 450, 450, 450, 540, 235, and 540 nm, respectively.

### 4.9. qRT-PCR Analysis

TRIzol^®^ reagent (Invitrogen, Carlsbad, CA, USA) was used to extract total RNA. Then, 5 µg of total RNA was collected and treated with DNase I for DNA digestion. The product was used to create transcribed complementary DNA (cDNA) with M-MLV (Tiangen, Beijing, China). Next, the expression levels of the identified genes were evaluated using the cDNA as a template. Primer Premier 5, version 5.0 (Premier Biosoft International, Palo Alto, USA) software was used to design primers; the primer sequences are presented in Table S1. The complete name, function, pathway, and classification of the detected genes are listed in Table S2. The online database Phytozome v13 (https://phytozome-next.jgi.doe.gov/ (accessed on 1 June 2023)) was used to obtain the above sequence and annotation information of the genes. The expression level of *SlACTIN* was used as an internal reference. The expression level of each gene was determined using the comparative *C_T_* method. The expression values were the averages of 3 biological duplicates.

### 4.10. Statistical Analysis

Data analysis was conducted using Excel 2016, GraphPad Prism 8, version 8.2.1 (GraphPad Software, San Diego, CA, USA), and SPSS, version 24.0 (SPPS Inc. Chicago, IL, USA). All data from three biological replicates were used. The Shapiro–Wilk and Levene’s tests were used to assess the normality and homoscedasticity of the data, and the significant differences was detected via a one-way ANOVA. To establish the statistical significance of the findings among distinct treatments, the *p* < 0.05 level was applied, and Duncan’s multiple range test was utilized for executing multiple comparisons.

## 5. Conclusions

The present study has demonstrated that during storage, the inhibition of SlGSNOR and the addition of NO synchronously delayed tomato fruit coloring and softening, and changed the accumulation of various nutrients, including soluble sugar; soluble protein; titratable acid; ascorbic acid; total phenol; and total flavonoid. Moreover, the repression of SlGSNOR and the addition of exogenous NO introduced more abundant endogenous NO than in untreated tomato fruit. The change of endogenous NO may be the main reason for triggering the accumulation of total carotenoid; lycopene; titratable acid; ascorbic acid; total phenol; and total flavonoid, and the decrease of chlorophylls; soluble sugar; and soluble protein. The molecular mechanism may be that SlGSNOR and NO inversely regulated the activity of chlorophyll synthesis and cell wall softening-related enzymes, and the transcripts of genes concerning pigment biosynthesis, softening, and nutrient metabolism. In summary, SlGSNOR plays a crucial role in postharvest tomato fruit ripening through regulating a variety of physiological and molecular alternations, which may be induced by its regulation in endogenous NO accumulation.

## Data Availability

All relevant data can be found within the paper and its Supplementary Data.

## Supplementary Materials

The following supporting information can be downloaded at https://www.mdpi.com/article/10.3390/ijms25052729/s1.
