# Supplementary material for: The Crucial Role of SlGSNOR in Regulating Postharvest Tomato Fruit Ripening
Source: Int J Mol Sci. 2024 Feb 27;25(5):2729. doi: 10.3390/ijms25052729 (PMC10932198; doi:10.3390/ijms25052729)
Supplement: Supplementary file 1 [file ijms-25-02729-s001.zip › ijms-2870118-supplementary.pdf]

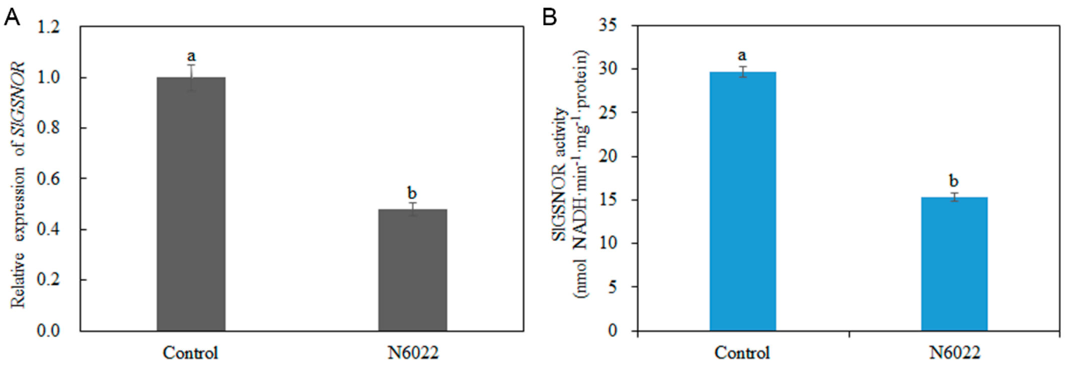

Figure S1. Effects of N6022 on *SlGSNOR* gene expression and SlGSNOR enzyme activity. (A) Expression of *SlGSNOR*; (B) SlGSNOR activity. Error bars represent standard deviations (n=3). Different lowercase letters denote statistically significant distinctions within different treatments according to Duncan's test ($p < 0.05$).

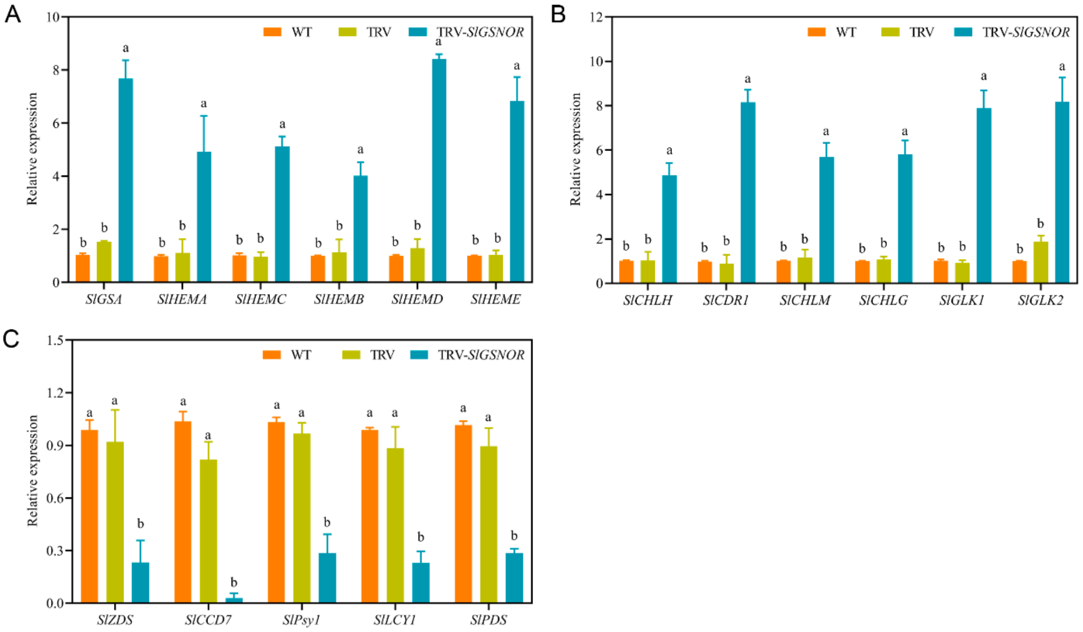

Figure S2. Effect of *SlGSNOR* silencing on pigment metabolism-related gene expressions. (A) Expressions of *SlGSA*, *SlHEMA*, *SlHEMC*, *SlHEMB*, *SlHEMD*, and *SlHEME*; (B) Expressions of *SlCHLH*, *SlCDR1*, *SlCHLM*, *SlCHLG*, *SlGLK1*, and *SlGLK2*; (C) Expressions of *SlZDS*, *SlCCD7*, *SlPsy1*, *SlLCY1*, and *SlPDS*. Error bars represent standard deviations (n=3). Different lowercase letters denote statistically significant distinctions within different treatments according to Duncan's test ($p < 0.05$).

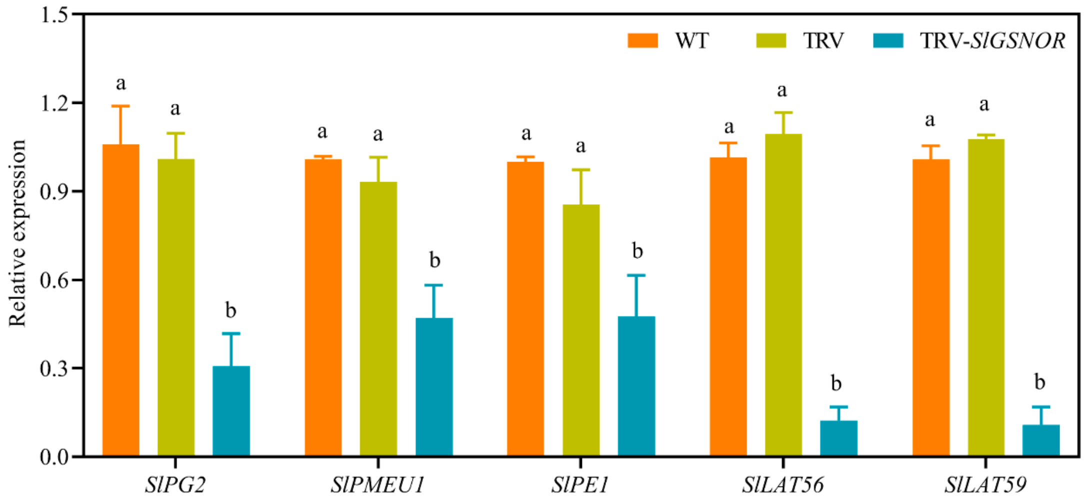

Figure S3. Effect of *SlGSNOR* silencing on hardness and texture related gene expressions. Expressions of *SlPG2*, *SlPMEU1*, *SlPE1*, *SlLAT56*, and *SlLAT59*. Error bars represent standard deviations (n=3). Different lowercase letters denote statistically significant distinctions within different treatments according to Duncan's test ($p < 0.05$).

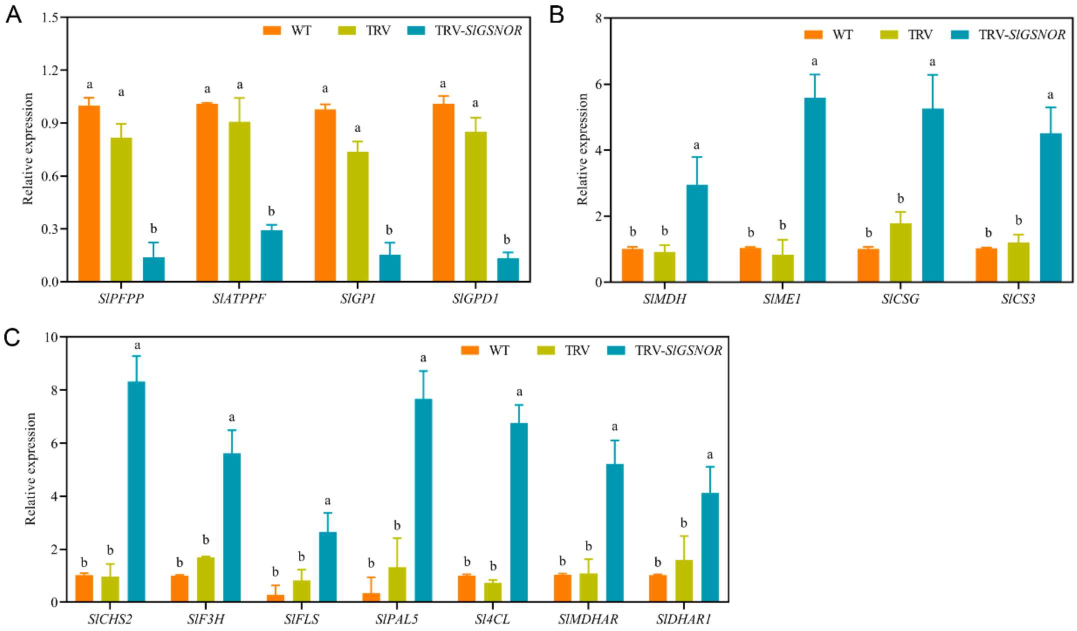

Figure S4. Effect of *SlGSNOR* silencing on nutrient related gene expressions. (A) Expressions of *SlPFPP*, *SlATPPF*, *SlGPI*, and *SlGPD1*; (B) Expressions of *SlMDH*, *SlME1*, *SlCSG*, and *SlCS3*; (C) Expressions of *SlCHS2*, *SlF3H*, *SlFLS*, *SlPAL5*, *Sl4CL*, *SlMDHAR*, and *SlDHAR1*. Error bars represent standard deviations (n=3). Different lowercase letters denote statistically significant distinctions within different treatments according to Duncan's test ($p < 0.05$).

Table S1. Primers used for qRT-PCR analysis.

Gene name	Gene ID	Primer sequence (5' to 3')	
SlACTIN	Solyc10g080500.2.1	F: AATGAACTTCGTGTGGCTCCAGAG	R: ATGGCAGGGGTGTTGAAGGTTTC
SlGSA	Solyc04g009200.3.1	F: CCATCTCACCGTCGATGCAA	R: TGGCATCAACTCCTTGGCTT
SlHEMA	Solyc04g076870.4.1	F: AACTTCTGCTGCTGATCGATATAC	R: CGACTGGTGCTGTGTGAATG
SlHEMC	Solyc03g098795.1.1	F: ACCTGCAGATCCGTTTCCTG	R: GGCAGCGGCAAGAATAATGG
SlHEMB	Solyc08g069030.4.1	F: ATGGTCTTGTTGAAGAGGTC	R: TATCAGCTCCTTCAGACTCA
SlHEMD	Solyc04g079320.4.1	F: TACTCGGCTGAACACTTACACAACG	R: TGCTACAGTAACAACGGGAACAGAG
SlHEME	Solyc10g007320.3.1	F: GCTTGTTTGTGGAATTAGGG	R: AAAATTGCAGCTTGTTCTCC
SlCHLH	Solyc04g015750.3.1	F: GCTTTGGACCCACAGGCTAT	R: CTGTGCCAACGACTCTCCAT
SlCDR1	Solyc08g005830.1.1	F: CCATCGTGATTCTCCTCTTT	R: ACATTACCACCACCTAAACC
SlCHLM	Solyc03g118240.4.1	F: ACCTTAAACCCTAATCCCCAAC	R: TGCCGCCACTGAACTACCT
SlCHLG	Solyc09g014760.4.1	F: TTTCAACTACAACAACCCACTTCG	R: CGTTTCTTGCTTGGCTCCTTT
SlGLK1	Solyc07g053630.4.1	F: GAGAAGTGGAGGCAGCGAGTTG	R: GAGAAGTGGAGGCAGCGAGTTG
SlGLK2	Solyc10g008160.3.1	F: AGCTTATGGCAACTCATGGTCTCAC	R: CGATTGTGGTGGCTCCGCTATAC
SlZDS	Solyc01g097810.3.1	F: CGGTTATTCGGGCTGATTTG	R: CCAAATCAGCCCGAATAACC
SlCCD7	Solyc01g090660.3.1	F: CCACGTCCCCAACTCATGAA	R: CTTCCACGACCCGAAGTGAA
SlPsy1	Solyc03g031860.3.1	F: TTATGTTGCTGGTACGGTTGGGTTG	R: CGCTCTCTGTTGTTGCCTTTGATTC
SlLCY1	Solyc04g040190.1.1	F: GTTGTTGTGGATCTTGCTGTGGTTG	R: AGAGAGTCCTGCTTCAGAAACTTGC
SlPDS	Solyc03g123760.3.1	F: TTATCTCGCAAGTGTGGCTATGGTG	R: GAAGATGCCTGCTTTCGGTGATTTG
SlPG2	Solyc10g080210.2.1	F: TGGAGGAGGAGGAACTATCAATGGC	R: GTTAAGGCCGTTGGTGCATCCC
SlPMEU1	Solyc03g123620.4.1	F: AAGCCGTTCGACGCAACTACTATG	R: AGTGTCTCGTCCATCGTCTCCAG
SlPE1	Solyc07g064190.2.1	F: CCCGCTAAAGCTATGCCGTTCAC	R: CACATACGCCACGCCAGTAGAC
SlLAT56	Solyc03g058910.3.1	F: GCACTCGGGACAGCAATAAA	R: GTCACATCTCCAGCACTTGTC
SlLAT59	Solyc03g058890.4.1	F: ATTAGGTTGCACCAGGAGATG	R: GAGGGTTATACCAGCACCTTTAG
SlPFPP	Solyc02g081160.3.1	F: GAGATCCACACGGCAATGTCCAG	R: CCTTCTGCTTCCTCTGATCCAACTC
SlATPPF	Solyc08g066100.3.1	F: CACGGCTTCTTGCTTCAACCAATC	R: CTTTGTGAGTGCTGTCGCTTATGC
SlGPI	Solyc12g014380.3.1	F: CATTCGCTTTCTGGGACTGGGTAG	R: ATGGTGCTGAGAGGAAATGCTGATC
SlGPD1	Solyc02g093830.4.1	F: GCCGTATTCGTGGGTATCTTTCTCG	R: TTCGTGCTCAGATATTGCCTTGTCC
SlMDH	Solyc11g007990.2.1	F: ATGAGGTGTTCCAGGATGCAGAATG	R: TCCAATAAGCCAGCTCGTTCCATAC
SlME1	Solyc12g044600.3.1	F: TCCCTCCTCTCATCCTCCCTCTC	R: CCCAGCTCCAGAAGATCCAATCAAG
SlCSG	Solyc12g011000.2.1	F: ACAGCATTCAGCAGTTCCACAAGG	R: AAGCATTAGCGGGTGACGAATCTG
SlCS3	Solyc01g073740.4.1	F: GTCTTTCTCCGCCTCTGTCTGTTTG	R: GCTCGGCAATATGGGATCGTGAC
SlCHS2	Solyc05g053550.3.1	F: GCCGACTACCAACTCACCAAGC	R: TCCCACCAGCAAAGCAACCTTG
SlF3H	Solyc02g083860.3.1	F: GAGGCAATGGGCTTAGAGAAAGAGG	R: GGTAATGGTTCCTGGATCGGTGTG
SlFLS	Solyc11g013110.2.1	F: ACCTTGAATGAGTCGGTGCTTGTG	R: GAGCCAGATTGTGGTCAGGTGTTC
SlPAL5	Solyc09g007910.2.1	F: GCCATCTAATCTCACAGCAGGAAGG	R: AAGTTCCGAGCAGTAAGAAGCCATC
Sl4CL	Solyc03g117870.3.1	F: ACCGCCTATTGTTCTCGCCATTG	R: GCTCGGACGGCATCTTCAAGTTC
SlMDHAR	Solyc08g081530.3.1	F: TGCTTCTCCACCATAACCCATTGC	R: GCGCGAAAGTTCAACAAGATCAGTG
SlDHAR1	Solyc05g054760.4.1	F: AAGAAGTGGAGTGTGCCTGAAAGC	R: CACGCATACAAGGACACGGTGAG
SlGSNOR	Solyc09g064370.4.1	F: ATGGCTACACAAGGTCAA	R: GTCCTGGATATGTTTGTATGA

Table S2. The complete name, function, pathway, and classification of the detected genes.

Gene name	Complete name	Function	Pathway	Gene classification
SlGSA	glutamate 1-semialdehyde 2,1-aminomutase	enables glutamate-1-semialdehyde 2,1-aminomutase activity	involved_in tetrapyrrole biosynthetic process that related to chlorophyll biosynthesis	
SlHEMA	glutamyl-tRNA reductase	enables glutamyl-tRNA reductase activity	involved_in tetrapyrrole biosynthetic process that related to chlorophyll biosynthesis	
SlHEMC	hydroxymethylbilane synthase	enables hydroxymethylbilane synthase activity	involved_in chlorophyll biosynthetic process	
SlHEMB	δ-aminolevulinic acid dehydratase	enables porphobilinogen synthase activity	involved_in heme biosynthetic process that related to chlorophyll biosynthesis	
SlHEMD	uroporphyrinogen-III synthase	enables uroporphyrinogen-III synthase activity	involved_in uroporphyrinogen III biosynthetic process that related to chlorophyll biosynthesis	
SlHEME	uroporphyrinogen decarboxylase	enables uroporphyrinogen decarboxylase activity	involved_in porphyrin-containing compound biosynthetic process that related to chlorophyll biosynthesis	
SlCHLH	magnesium-chelatase H subunit	enables magnesium chelatase activity	involved_in chlorophyll biosynthetic process	
SlCDR1	aspartic proteinase	enables aspartic-type endopeptidase activity	involved_in proteolysis process that related chlorophyll biosynthetic process	chlorophyll biosynthesis-related genes
SlCHLM	magnesium protoporphyrin IX methyltransferase	enables magnesium protoporphyrin IX methyltransferase activity	involved_in chlorophyll biosynthetic process	
SlCHLG	chlorophyll synthase	enables chlorophyll synthetase activity	involved_in chlorophyll biosynthetic process	
SlGLK1	golden1-like protein 1	enables DNA-binding transcription factor activity	involved_in positive regulation of DNA-templated transcription that related to chlorophyll biosynthesis	
SlGLK2	golden1-like protein 2	enables DNA-binding transcription factor activity	involved_in positive regulation of DNA-templated transcription that related to chlorophyll biosynthesis	
SlZDS	ζ-carotene desaturase	enables 9,9'-di-cis-ζ-carotene desaturase activity	involved_in carotenoid biosynthetic process	
SlCCD7	carotenoid cleavage dioxygenase 7	enables 9-cis-epoxycarotenoid dioxygenase activity	involved_in carotene catabolic process	
SlPsy1	phytoene synthase 1	enables phytoene synthase activity	involved_in carotenoid biosynthetic process	
SlLCY1	lycopene β-cyclase 1	enables oxidoreductase activity, acting on paired donors, with incorporation or reduction of molecular oxygen	involved_in carotenoid biosynthetic process	
SlPDS	phytoene dehydrogenase	enables phytoene dehydrogenase activity	involved_in carotenoid biosynthetic process	

SlPG2	polygalacturonase-2a	enables polygalacturonase activity	involved_in pectin catabolic process	
SlPMEU1	pectin methylesterase	enables pectinesterase activity	involved_in cell wall modification	
SlPE1	pectinesterase 1	enables pectinesterase activity	involved_in cell wall modification	fruit softening-related genes
SlLAT56	pectate lyase P56	enables pectate lyase activity	involved_in cell wall degradation and fruit softening	
SlLAT59	pectate lyase P59	enables pectate lyase activity	involved_in cell wall degradation and fruit softening	
SlPFPP	pyrophosphate-fructose 6-phosphate 1-phosphotransferase subunit β-like	enables diphosphate-fructose-6-phosphate 1-phosphotransferase activity	involved_in fructose 6-phosphate metabolic process	
SlATPPF	ATP-dependent 6-phosphofructokinase 6	enables 6-phosphofructokinase activity	involved_in fructose 6-phosphate metabolic process	
SlGPI	glucose-6-phosphate isomerase	enables glucose-6-phosphate isomerase activity	involved_in glucose 6-phosphate metabolic process	
SlGPD1	glucose-6-phosphate 1-dehydrogenase 1	enables glucose-6-phosphate dehydrogenase activity	involved_in glucose metabolic process	glucose and fructose metabolism-related genes
SlMDH	malate dehydrogenase	enables L-malate dehydrogenase activity	involved_in malate metabolic process	
SlME1	NADP-malic enzyme	enables malate dehydrogenase (decarboxylating) (NAD+) activity	involved_in malate metabolic process	
SlCSG	citrate synthase, glyoxysomal	enables citrate (Si)-synthase activity	involved_in carbohydrate metabolic process	
SlCS3	citrate synthase 3	enables citrate (Si)-synthase activity	involved_in carbohydrate metabolic process	
SlCHS2	chalcone synthase	enables chalcone synthase activity	involved_in flavonoid metabolism process	
SlF3H	flavanone 3-hydroxylase	enables flavanone 3-hydroxylase activity	involved_in flavonoid metabolism process	flavonoid metabolism-related genes
SlFLS	flavonol synthase	enables flavonol synthase activity	involved_in flavonoid metabolism process	
SlPAL5	phenylalanine ammonia-lyase 5	enables ammonia-lyase activity	involved_in phenol metabolism process	phenol metabolism-related genes
Sl4CL	4-coumarate-CoA ligase	enables CoA-ligase activity	involved_in phenol metabolism process	
SlMDHAR	monodehydroascorbate reductase	enables oxidoreductase activity, acting on NAD(P)H	involved_in ascorbic acid metabolism process	ascorbic acid metabolism-related genes
SlDHAR1	dehydroascorbate reductase 1	enables glutathione dehydrogenase (ascorbate) activity	involved_in ascorbate glutathione cycle	
SlGSNOR	*S*-nitrosoglutathione reductase	enables *S*-nitrosoglutathione reductase activity	involved_in reduction of protein *S*-nitrosylation	protein *S*-nitrosylation reduction-related gene
